# Elimination of reserve cells for prevention of HPV-associated cervical cancer

**DOI:** 10.1016/j.virusres.2023.199068

**Published:** 2023-03-16

**Authors:** Olaf Reich, Sigrid Regauer

**Affiliations:** a Department of Obstetrics & Gynecology , Medical University of Graz, Graz, Austria; b Diagnostic & Research Institute of Pathology, Medical University of Graz, Graz, Austria

**Keywords:** HPV, HPV research model, Reserve cells, Cervical carcinogenesis, Treatment, Recurrent disease, Prevention

## Abstract

Human papilloma viruses (HPV), that are causative for most squamous cell cervical cancers (SCC), have a simple structure with only a few genes (six early and two late genes). Two of the early HPV genes (E6 and E7) are capable of transforming normal squamous epithelium into cancer. In the last 10 years, a controversial discussion arose as to which cells are primarily involved in cervical carcinogenesis. Virologists traditionally use a research model of stratified squamous epithelium, a permissive environment for completion of a full HPV-life cycle. Basic insights on HPV tropism, HPV life cycle, HPV-uptake, HPV-replication, HPV-gene expression were gained from this model. Stratified squamous epithelium, however, is a low-risk area for SCC. Most SCC develop in an area of endocervical columnar epithelium that undergoes squamous metaplasia. SCC arise after infection of immature squamous metaplasia, proliferating reserve cells/reserve cell hyperplasia and reserve cells of the endocervical columnar epithelium. Study models investigating this pathway of carcinogenesis do not exist and therapeutic consequences deduced from this knowledge are lacking. This review describes in detail cervical carcinogenesis after HPV infection of subcolumnar reserve cells and discusses new intervention strategies for patients. The WHO-launched global strategy to eliminate HPV-associated cervical cancer builds primarily on prophylactic vaccination, screening and treatment. New insights in cervical pathogenesis, may assist in reaching this ambitious WHO goal.

## 1 Introduction

Human papilloma viruses (HPV) are small DNA viruses that replicate in specific anatomical sites. They have a simple structure and are built of only a few genes (six early and two late genes). The late genes (L1 and L2) encode the major capsid proteins. The early genes (E6, E7, E1, E2, E4, E5) are involved in replication control. Two of the early HPV genes (E6 and E7) are able to transform normal squamous epithelium into cancer due to their ability to immortalize cells. Virologists traditionally use a research model of stratified squamous epithelium, a permissive environment for completion of the HPV-life cycle. Basic insights on HPV associated disease including viral tropism, viral life cycle, HPV-uptake, HPV-replication, HPV-gene expression, the concept of transforming HPV infection in the development of HPV-associated squamous cell cancers (SCC) and immune control of HPV-infection are based on a study model consisting of this stratified squamous epithelium.

In fact, this “*stratified squamous epithelium model*” is suitable for research on HPV-associated carcinogenesis in stratrified squamous epithelium of the skin, penis, vulva, vagina, and oral cavity. By contrast, this model has short comings when explaining cervical carcinogenesis, because most cervical SCC originate in an area initially covered by non-squamous endocervical epithelium consisting of tall columnar epithelium with subcolumnar reserve cells capable of squamous metaplasia. The area in which most cervical SCC develop is called **transformation zone**, and features several maturation levels of metaplastic squamous epithelium bordering tall columnar epithelium with subcolumnar epithelium.

**Fig. 1:**
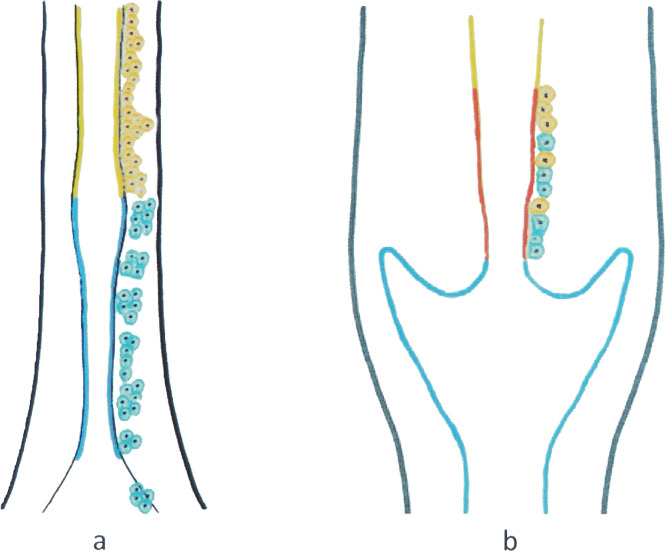
Overview of the prenatal development of uterine cervix (adapted from: Fritsch H., et al., The development of the human vaginal fornix and the portio cervicis. Clin. Anat. 2020; 34:1059-1067). Fig. 1a Early embryologic development: Two different cell lineages meet at the utero-vaginal anlagen. Schematic drawing indicating the border of urogenital sinus structures (blue) and Müllerian structures (yellow). Fig. 1b Late prenatal development: Schematic drawing indicating endocervical epithelium capable of squamous metaplasia (red line) with urogenital sinus derived Ck17 positive reserve cells (blue) and Müllerian derived Ck7 positive reserve cells (yellow).

**Fig. 2:**
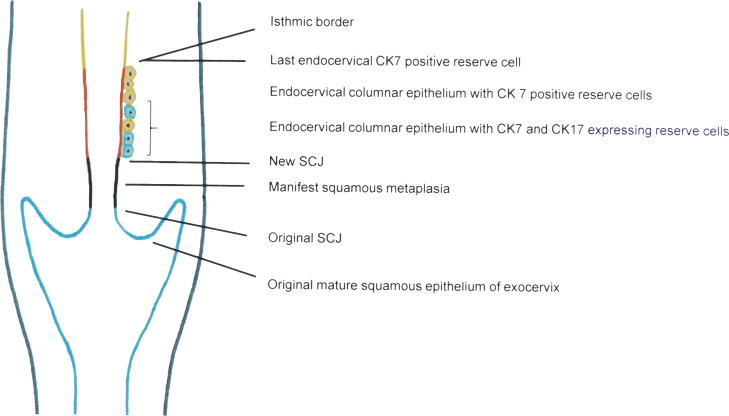
Overview of the epithelial situation of the adult uterine cervix (adapted from: Fritsch H., et al., The development of the human vaginal fornix and the portio cervicis. Clin. Anat. 2020; 34:1059-1067). Blue line: The vagina, the fornices and the ectocervix are covered by a thick stratified mature glycogenated squamous epithelium referred to as original squamous epithelium. The original squamous epithelium is a low-risk area for squamous cervical cancer. Black line: Manifest metaplastic squamous epithelium of the transformation zone resembling the original squamous epithelium. It is derived from p63/CK17-positive reserve cells. As glycogenated squamous epithelium (original or metaplastic) represents a permissive environment for completion of a full HPV-life cycle, this area is also a low-risk area for squamous cervical cancer. By contrast, endocervical tall columnar epithelium with subcolumnar reserve cells with beginning immature squamous metaplasia is a high-risk area for squamous cervical cancer. A transforming HPV-infection of proliferating reserve cells and HPV-transformed immature metaplastic squamous epithelium is characterized by high-level expression of E6 and E7 HPV genes. These developing high-grade intraepithelial lesions are thin full-thickness lesions up to 9 cell layers in thickness. Red line: Endocervical columnar epithelium with urogenital sinus derived p63/CK17-positive reserve cells (blue cells) and Müllerian-derived CK7-positive reserve cells (yellow cells). These reserve cells may serve as reservoir of HPV infection. A transforming HPV infection of these (residual) reserve cells are causal for recurrent HSIL after destruction / treatment of HSIL. Yellow line: Isthmic mucosa as part of Müllerian derived endometrium. The last CK7 positive endocervical reserve cell represents the upper border of the uterine epithelium that is sensitive for HPV.

In the last 10 years a controversial discussion arose as to which cells are primarily involved in cervical carcinogenesis. It is well accepted that reserve cells give rise to metaplastic squamous epithelium and that high-grade squamous intraepithelial lesions (HSIL)/SCC develop after HPV-infection of metaplastic squamous epithelium of different maturation levels. Reserve cells develop from two different embryonic structures: Urogenital sinus derived p63/CK17-positive cells are the primary precursor cells for the squamous epithelium/HSIL/SCC, and Müllerian-derived CK7-positive reserve cells for the columnar epithelium/adenocarcinoma in situ (AIS)/adenocarcinoma (AC). Reserve cells extend from the squamocolumnar junction (SCJ) throughout the entire cervical columnar epithelium to the isthmic border (Fritsch et al., 2021; Regauer et al., 2022) (Figs. 1 and 2). These observations contradict claims that HSIL/SCC only emerge from a few distinct epithelial cells at the SCJ (Herfs et al., 2012, 2015). Pathologists involved in every day surgical histopathological practise readily recognize the complex situation of the cervical mucosa and the various maturation levels of squamous epithelium and proliferating reserve cells in cone specimens. Quite often proliferating reserve cells and / or immature squamous metaplasia is present at the endocervical resection margin. Despite correct treatment according to current guidelines, recurrent HSIL after removal of the SCJ and portions of squamous metaplasia with the HSIL remain a problem. Recurrences may arise from mature squamous epithelium, but also are linked to residual subcolumnar reserve cells in the endocervical canal. The complex situation of the human cervix during active transformation present virologists and gynecologists with specific problems: Virologists need to extend their model and concept of HPV-associated disease, and gynecologists need to discuss modification of treatment regimes, that include not only treatment of a lesion, but – at the same time – also prevents recurrences. This critical review on the origin of cervical SCC is based on published observational embryological, fetal and adult studies since fate determining human embryological studies do not exist.

## 2 Epidemiological aspects of cervical pre/cancer

Cervical cancer is one of the most common causes of cancer deaths in women globally. In 2020, the WHO launched a global strategy to accelerate the elimination of cervical cancer that focusses on prophylactic vaccination, screening and treatment of women at risk for invasive disease (Canfell, 2019). The risk of cervical cancer varies widely among different population groups, with the highest risk seen in persons living with the human immunodeficiency virus (HIV) (Abraham et al., 2013). In low- and middle-income countries ablative techniques (eg. cryosurgery, electrocautery, cold coagulation) are used to treat cervical cancer precursors in see and treat programs. In developed countries excisional techniques (eg., large loope excision of the transformation zone (LLETZ), loop diathermy conization, laser conization or cold-knife conization) are the standard of care for both HSIL and AIS.

## 3 Current problems in the management of cervical precancer

Retrospective analyses show that women with treated HSIL are at increased risk of recurrent HSIL and even invasive squamous cervical cancer (SCC) (Arbyn et al., 2017; Brown et al., 1991; Guido et al., 2014; Martin-Hirsch et al., 2013; Kocken et al., 2011; Kalliala et al., 2020; Sand et al., 2018; Sand et al., 2022; Soutter et al., 2006; Strander et al., 2007; Strander et al., 2014; van de et al., 2022; van der Heijden et al., 2015; Kashofer et al., 2023). Following excision of HSIL using LLETZ, recurrent HSIL rates range from 4% to 27% (Guido et al., 2014; Martin-Hirsch et al., 2013; van der Heijden et al., 2015). One third of women with two LLETZ procedures require an additional excisional procedure or hysterectomy (van de et al., 2022). The risk of invasive SCC after excisional treatment for HSIL is five times higher than in the general population (Brown et al., 1991; Soutter et al., 2006), and it increases with HPV-persistence, involved margins, small excision sizes, age, and immunosuppression/ immunodeficiency (Arbyn et al., 2017; Strander et al., 2014; van der Heijden et al., 2015). There obviously is a need of becoming better in the prevention of recurrent HSIL/AIS and invasive disease. This paper presents a proposal of a targeted intervention strategy for patients at risk for cervical pre/cancer will be discussed to stimulate interdisciplinary cross talk between scientists, clinicians, guidelines commissioners, screening providers and program managers.

## 4 Scientific rationale behind targeted intervention strategies

The observation that most cervical precancerous lesions develop within the area of squamous metaplastic epithelium was an important milestone in understanding cervical oncogenesis and is credited to Hans Hinselmann (1884–1959). He presented a completely new view of cervical oncogenesis based on his colposcopic and histologic findings. He described two different types of cervical squamous epithelia. The first type was the (preexisting) original squamous epithelium, and the second type was a newly formed squamous metaplastic epithelium. He called this zone (area) of metaplastic epithelium “Umwandlungszone” (Hinselmann, 1927). This sentinel publication was in German language. Much later the term “Umwandlungszone” was translated as transformation zone (TZ). Colposcopist throughout the world use the term TZ for an area were squamous metaplasia is in progress, and quite often refer to the area of manifest metaplasia (Reich et al., 2017). The original SCJ between original squamous epithelium and metaplastic squamous epithelium is the outer border of the TZ and remains a landmark throughout life. The new SCJ is found between the expanding metaplastic squamous epithelium and the endocervical columnar epithelium, and is typically a well developed circumferential step-like border (Reich et al., 2017). The newly formed area/zone of metaplasia is complex, as fully matured metaplastic epithelium co-exists with still immature squamous epithelium. During active squamous metaplasia small islands of immature metaplastic squamous epithelium may interdigitate with endocervical columnar epithelium, and small islands of immature squamous metaplastic epithelium appear within endocervical columnar epithelium. The concept of squamous metaplasia is central to the understanding of the pathogenesis of HSIL/SCC because the maturation level of squamous epithelium at time of HPV infections determines the cancer risk (Doorbar and Griffin., 2019; Doorbar et al., 2021; Regauer and Reich, 2021; Regauer and Reich, 2007; Regauer et al., 2019; Regauer et al., 2022; Reich and Regauer., 2014; Reich and Regauer, 2017; Reich and Regauer, 2019. Mature metaplastic (glycogenated) squamous epithelium has the same low risk for HSIL/SCC than the original squamous epithelium of cervix and vagina (Burghardt, 1976; Regauer and Reich, 2007; Regauer et al., 2019; Regauer and Reich, 2021; Regauer et al., 2022; Reich and Regauer, 2014; Reich and Regauer., 2017; Reich and Regauer, 2019). The high-risk area, however, where most HSIL/SCC arise, corresponds to the area where (proliferating) reserve cells and immature metaplastic squamous epithelium are easily accessible for HPV (Burghardt, 1976; Doorbar and Griffin, 2019; Doorbar et al., 2021; Regauer and Reich, 2007; Regauer et al., 2019; Regauer and Reich, 2021; Regauer et al., 2022; Reich 2010; Reich and Regauer, 2017; Reich and Regauer, 2019). Two types of reserve cells of different embryonic origin and in prenatally determined distribution are capable of squamous metaplasia, and both can be identified throughout the entire endocervical columnar epithelium (Fritsch et al., 2021; Martens et al., 2009; Regauer and Reich, 2021) (Fig. 1). Reserve cells of urogenital sinus (UGS) origin, characterised by p63/CK17 co-expression, are concentrated in endocervical columnar epithelium near the SCJ and serve as primary target cells for squamous metaplasia. During second trimester growth and remodelling, they migrate towards the cervix and reach their final location in the last trimester. The second type of reserve cells expresses CK7 and is of Müllerian origin. They serve as stem cells for columnar cells and AIS. These reserve cells have stem cell character as they have also the capacity to acquire CK17/p63 expression and to develop into squamous metaplastic epithelium, (recurrent) HSIL, and SCC (Regauer and Reich, 2021). Based on this knowledge, it is a robust argument to use the term TZ for the entire zone/area where squamous metaplasia can occur and has occurred (Fig. 2). Colposcopically, however, only the area of manifest squamous metaplasia can be appreciated (Regauer and Reich, 2021; Reich et al., 2017).

One of the current misconceptions about stem cells of cervical cancer is based on recent studies on embryos and fetal tissues before development of adult-type epithelia and before definitive positioning of reserve cells has occured. This theory postulates that cuboidal stem cells at the SCJ identified by the “stem cell marker” CK7 give rise to HSIL/SCC (Herfs et al., 2012). However, CK7 also expressed in the Müllerian derived reserve cells, and in the entire endocervical columnar epithelium (Moll et al., 1983; Ramaekers et al., 1987; Regauer and Reich, 2021; Smedts et al., 1993). The short segments of CK7-positive cells at the SCJ are adapted endocervical columnar cells that accommodate for the anatomical difference in epithelial thickness between tall columnar single layered endocervical epithelium and thick stratified (metaplastic) squamous epithelium. Their presence has been acknowledged for more than half of a century, and these modified endocervical columnar cells have no significance for cervical carcinogenesis (Bajardi1962).

## 5 Treatment recommendations of the 2011 IFCPC consensus meeting

Current recommendations for HSIL/AIS treatment are based on expert opinions and lack rigorous evidence-based support. In 2011, the IFCPC approved a revision of colposcopic terminology (Bornstein et al., 2012). To standardize treatment, three types of TZ (defined as manifest squamous metaplasia) were specified based on extent of squamous metaplasia and the visibility of the new SCJ (upper limit of the metaplastic squamous epithelium) (Fig. 2). Position and visibility of the new SCJ should aid surgeons in the attempt of complete eradication of a HSIL. In a type 1 TZ, a lesion is located completely in ectocervical position. In a type 2 TZ, the lesion has an endocervical component but the new SCJ is fully visible at colposcopy. In type 3 TZ, a lesion has an endocervical component, and the upper limit of the new SCJ is not or not fully visible. Lesions in type 1 and type 2 TZ may be treated with either destructive or excisional techniques. Excison is mandatory in patients with type 3 TZ, and destructive methods should not be used.

## 6 Adapting extent of treatment to prevent recurrent HSIL/SCC

The risk of recurrent or new HSIL post-treatment mainly depends on presence of residual endocervical columnar epithelium. CK17/p63 and / or CK7 expressing reserve cells will undergo new squamous differentiation, and thus are ultimately responsible for new immature/mature squamous metaplasia, recurrent HSIL and SCC (Regauer and Reich, 2021; Reich and Regauer, 2015). This explains why resection of HSIL only can not prevent recurrences, even if the resection margins are clear (Debeaudrap et al., 2019; Paraskevaidis et al., 2000; van der Heijden et al., 2015). This observation led to the concept of the last endocervical reserve cells, which was first introduced in studies of prenatal development of the cervix (Fritsch et al., 2021, Regauer and Reich, 2021) (Fig. 2). The last endocervical reserve cell is a biologic landmark that denotes the inner limit of endocervical columnar epithelium at risk for HSIL/SCC. However, it can not be identified with the colposcope. Furthermore, the anatomic position of the last endocervical reserve cell is inconsistent as it shifts during different life cycle of a woman (Schneppenheim et al., 1958). Clinical observations corroborate this concept, as removal of HSIL including a large portion of “normal” endocervical columnar epithelium reduced recurrence rate to near 0% (Reich et al., 2001). It is of global relevance that only effective elimination of reserve cells of endocervical columnar epithelium will prevent development of new squamous metaplasia and recurrent HSIL/SCC, but the 2011 IFCPC recommendation has not addressed these facts (Bornstein et al., 2012).

Countries with limited medical access: Women in low income and middle income countries have the highest risk of cervical cancer/cervical cancer deaths (Sahasrabuddhe et al., 2012). The highest incidence of cervical cancer is found in Sub-Saharan Africa, especially in Southern and Eastern Africa, which in 2018 had an age-standardized incidence rate of 43.1 and 40.1 per 100,000 women, respectively (de Martel et al., 2020). Infections with HIV have reached epidemic proportions in Afrika. HIV-infected women have a high prevalence of persistent HPV infection between 40%-59% (Swai et al., 2022). Numerous studies have clearly documentated an association between latent HIV infection and cervical pre/cancer. Women living with HIV have a six-fold higher risk of developing cervical cancer compared to women without HIV infection (Abraham et al., 2013; Swai et al., 2022). To address the high burden of HPV-HIV coinfection with the increased risk for HSIL/SCC, a *Prophylactic Ablation* of the area at risk for HSIL/SCC could become a novel standard of care for women living with HIV (Taylor et al., 2010). The protective oncologic effect from electrocautery of the ectocervix post partum has been acknowledged since 1957 (Younge, 1957).

Scientific studies evaluating the outcomes after prophylactic ablation are rare, but in great demand. The erroneous claim of embryonic cells at the SCJ led to proposals of prophylactic ablationof the new SCJ in resource-limited settings (Franceschi, 2015a,b; Fritsch et al., 2021; Herfs et al., 2015; Herfs and Crum, 2015). A true prophylaxis of HSIL/SCC, however, needs ablation of the entire endocervical columnar epithelium harbouring reserve cells (Regauer and Reich, 2021). Randomized controlled trials will tell if prophylactic ablation of the metaplastic squamous epithelium and reserve cells of endocervical columnar epithelium protects women better from HSIL/SCC than current management (Regauer and Reich, 2021).

Developed countries: The use of excision techniques is the mainstay in the management of HSIL in developed countries. LLETZ is a simple procedure, but the central question concerns the size of an excison which impacts on both, obstetrical and oncological outcomes (Kocken et al., 2011; Kyrgiou et al., 2011; Reich et al., 2001; Strander et al., 2007). During the past decades, excisional procedures at the cervix became less radical because obstetric adverse events increase with cone dimensions, while at the same time, recurrent HSIL were up to 90 times more frequent (Reich et al., 2001; van der Heijden et al., 2015). For women with HSIL wishing to retain fertility, a *Limited Excision Procedure* avoids unnecessary destruction and loss of healthy cervical tissue. Cervical crypt involvement by HSIL usually does not exceed 5 mm and LEEP procedures remove tissues with the necessary depth (Anderson and Hartley, 1980; Taxa et al., 2018). Such a procedure, however, has no preventive function. Therefore, we propose an *Extended Excisional Procedure* that moves away from the current standard of treating solely the lesion in women beyond reproductive age. Based on the concept of the last endocervical reserve cell, the *Extended Excisional Procedure* calls for additional removal of healthy endocervical columnar epithelium with reserve cells. The endocervical length of excision should be approximately 15 mm and may be carried out by LEEP, needle excision, or cold knife. Electrocautery / laser coagulation of the wound surface assists in controlling bleeding and destroys an additional 5 mm of columnar epithelium at the endocervical margin (Reich et al., 2000). Trained surgeons should be able to manage complications after larger excisional treatments such as increased risk of postoperative bleeding and discharge.

## 7 Adenocarcinoma in situ (AIS)

AIS originates from CK7 expressing reserve cells of Müllerian origin, the stem cell for glandular lesions and renewal (Fritsch et al., 2021; Regauer and Reich, 2021). Colposcopists are unable to determine the limits of AIS. Hence, the IFCPC recommends a type III excision. In patients with a fully visible new SCJ, BSCCP and the Society of Gynecologic Oncology (SGO) advise an excision containing at least 10 mm of the endocervical canal (Bornstein et al., 2012, Teoh et al., 2020). In patients without a visible new SCJ, an excision should have an endocervical length of 20 to 25 mm (https://www.gov.uk/government/publications/cervical-screening-programme-and-colposcopy-management). Positive margins, however, are reported in 44% after LEETZ and 29% after cold knife conization requiring re-excision or hysterectomy (Jiang et al., 2017; Ritu, 2009). Regarding the ectocervical incision line, the IFCPC and SGO gave no recommendation. A complete removal of the entire glandular field below manifest mature squamous metaplasia calls for a resection beginning at the last cervical gland at the original SCJ with a circumferential incision that extends into the endocervical canal for a length of 15-25 mm. In case the original SCJ cannot be adequately visualized (gland operings and ovula nabothi are absent), the resection should start at the peripheral third of the ectocervix. In women who want to become pregnant after such a procedure, laparoscopic cerclage is a highly effective option for cervical insufficiency after large excisions (Clark and Einarsson, 2020).

## 8 Summary and perspective

Prophylactic vaccines and screening programmes have successfully reduced the burden of HPV infections and cervical pre/cancer. HPV vaccination, however, does little for those women who are infected by non-vaccine HPV types. Current efforts in management focus on treatment of the precancerous lesions only without offering prevention of recurrent and invasive disease post-treatment. Treatment of the whole area of risk for HSIL/SCC with targeted intervention strategies opens the way for future reducing the incidence and mortality from cervical cancer.

## Data Availability

No data was used for the research described in the article.

## Glossary

AIS: is a glandular intraepithelial neoplasm (Mills et al., 2020).
Embryological stem cells: Squamous metaplasia and subsequent HSIL/SCC arise from reserve cells of 2 different embryological origins. The location and distribution of stem/reserve cells is prenatally determined. Both types of stem/reserve cells are not limited to an epithelial niche at the SCJ, rather, they are distributed throughout the entire endocervical columnar epithelium. While the CK17 / p63 co-expressing reserve cells are already destined for squamous differentiation, the CK7-expressing reserve cells of Müllerian origin have true stem cell character, as they are capable of glandular renewal, but may also differentiate into a squamous CK17 / p63 co-expressing stem cell for squamous metaplasia. The erroneous claim of a stem cell niche may be related to studies of fetal tissues before reserve cells had reached their final position and problematic interpretation of immunohistochemical staining results with antibody to CK7. All differentiated tall columnar endocervical cells stain with antibody to CK7. Therefore, CK7 is by no means a stem cell marker (Fritsch et al., 2021; Martens et al., 2009; Regauer and Reich, 2021) (Fig. 1).
High-risk area for squamous cervical cancer: The area of immature squamous metaplasia, proliferating reserve/reserve cell hyperplasia and the columnar endocervical epithelium with reserve cells. 90% of HSIL/SCC develop here (Burghardt, 1976; Regauer and Reich, 2021).
Immature squamous metaplasia: Metaplasia always begins with proliferation of reserve cells, partly still topped by tall endocervical columnar epithelium that later will be sludged off. It typically is composed of several layers of uniform cuboidal cells that lack glycogenated or cornified superficial cell layers (Mills et al., 2020).
Last endocervical reserve cell: is a biologic landmark identifying the upper border of endocervical columnar epithelium at risk for HPV-infection and subsequent HSIL/SCC (Regauer and Reich, 2021) (Fig. 2)
Low-grade squamous intraepithelial lesions: LSIL develops after a HPV infection of mature stratified squamous epithelium, which constitutes a permissive environment for completion of HPV life cycle. Typically, HPV-associated changes (koilocytosis) are observed. LSIL can develop after infection with low-risk- und high-risk HPV genotypes (Herrington et al., 2020).
Low-risk area for squamous cervical cancer: Mature stratified squamous epithelium, independent of origin, i.e. either original squamous epithelium in ectocervix and vagina, or of metaplastic origin. Only a minority of squamous cell pre/cancers arise here via LSIL (Burghardt, 1976; Regauer and Reich, 2021) (Fig. 2).
Mature squamous epithelium: is a stratified glycogenated squamous epithelium, that is either original (original squamous epithelium) or developed as result of metaplasia. Colposcopically, the mature metaplastic squamous epithelium of the TZ can be distinguished only from the mature original squamous epithelium by the presence of gland openings, more prominent blood vessels, or retention cysts. At both locations mature squamous epithelium constitutes a permissive environment for completion of HPV life cycle and assembly of virons (Herrington et al., 2020; Regauer and Reich, 2021) (Fig. 2).
Reserve cells: Two types of reserve cells can be identified within the endocervical columnar epithelium: p63/CK17 co-expressing reserve cells are of UGS origin. Tissue of UGS origin become located towards the cervix during fetal growth the second trimester. They reach their final location in the endocervical columnar epithelium in the last trimester and therefore, their post-natal / adult distribution is prenatally determined. The UGS derived reserve cells are the primary target cells for squamous metaplasia. The second type of reserve cells is of Müllerian origin, expresses CK7 and serves as stem cell for AIS and glandular renewal. These reserve cells have true stem cell character as they have the capacity to acquire CK17/ p63 expression. This type of reserve cells is responsible for formation of new squamous epithelium / HSIL / SCC after resection/ablation of HSIL with areas of TZ harboring CK17/ p63 reserve cells (Fritsch et al., 2021; Martens et al., 2009; Regauer and Reich, 2021) (Fig. 1,2).
Risk area for recurrent HSIL/SCC: Residual endocervical columnar epithelium harboring reserve cells, irrespective of embryologic origin. Residual reserve cells are capable of forming new squamous epithelium and subsequent HSIL/SCC after treatment of HSIL (Regauer and Reich, 2021; Reich and Regauer, 2015) (Fig. 2).
Squamo-columnar junction (SCJ): Is divided into original and new SCJ. In adolescence before menarche, the *original SCJ* is the border between the original squamous epithelium and the tall columnar epithelium. After metaplasia has occurred the newly formed border between immature or mature metaplastic squamous epithelium and the tall columnar epithelium is called *new SCJ*. The new SCJ is a critical hallmark for colposcopists when treating HSIL (Regauer and Reich, 2021; Reich at al. 2017) (Fig. 2).
Stem cell niche: The claim of an epithelial stem cell niche results from studies on fetal tissues before reserve cells have reached their final position, and was compounded by problematic interpretations of immunohistochemical staining results with antibody to CK7, which was falsely identified as “stem cell marker”. Reserve cells of two different embryological origins with prenatally determined location and distribution serve as stem cells for squamous metaplasia. They are distributed throughout the entire endocervical columnar epithelium, and not limited to an epithelial niche. The presumed stem cells at the SCJ are rather adapted CK7-positive endocervical columnar cells to accommodate for the difference of thickness of two types of epithelia (Fritsch et al., 2021; Martens et al., 2009; Regauer and Reich, 2021).
Thin HSIL: are high-grade squamous intraepithelial lesions ≤9 cell layers thick. They develop after HPV infections of reserve cells or immature squamous epithelium and are easily mistaken for immature metaplasia. Up to 30% of thin HSIL arise after infection with non-high-risk HPV (Herrington et al., 2020; Regauer and Reich, 2007; Regauer et al., 2019; Regauer et al., 2022; Reich and Regauer, 2017).
Thick HSIL: are high-grade squamous intraepithelial lesions ≥ 10 cell layers thick. They can develop after a transforming HPV infection via two pathways: i) either via LSIL after HPV infection of mature glycogenated squamous epithelium or ii) via thin HSIL after infection of reserve cells or immature metaplastic cells. HSIL developed through LSIL often still feature koilocytes (Herrington 2020; Regauer and Reich, 2021; Regauer et al., 2022; Reich and Regauer, 2014).
Transformation Zone (TZ): is the area of risk for SCC. It corresponds to the area where reserve cells capable of squamous metaplasia reside in the cervix. The TZ consist of the area where squamous metaplasia has already occurred (i.e. where tall columnar endocervical epithelium has been replaced by mature squamous metaplasia) and an area that is still covered by tall columnar endocervical epithelium with (proliferating) CK17 and CK7 expressing reserve cells that have the potential to undergo squamous metaplasia. Therefore, in the TZ, mature and immature metaplastic squamous epithelium co-exists with endocervical columnar epithelium that harbors reserve cells. Most colposcopists, however, use the term TZ to refer to the area of manifest squamous metaplastic epithelium between both SCJs (Regauer and Reich, 2021; Regauer et al., 2022; Reich et al., 2017) (Fig. 2).
